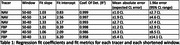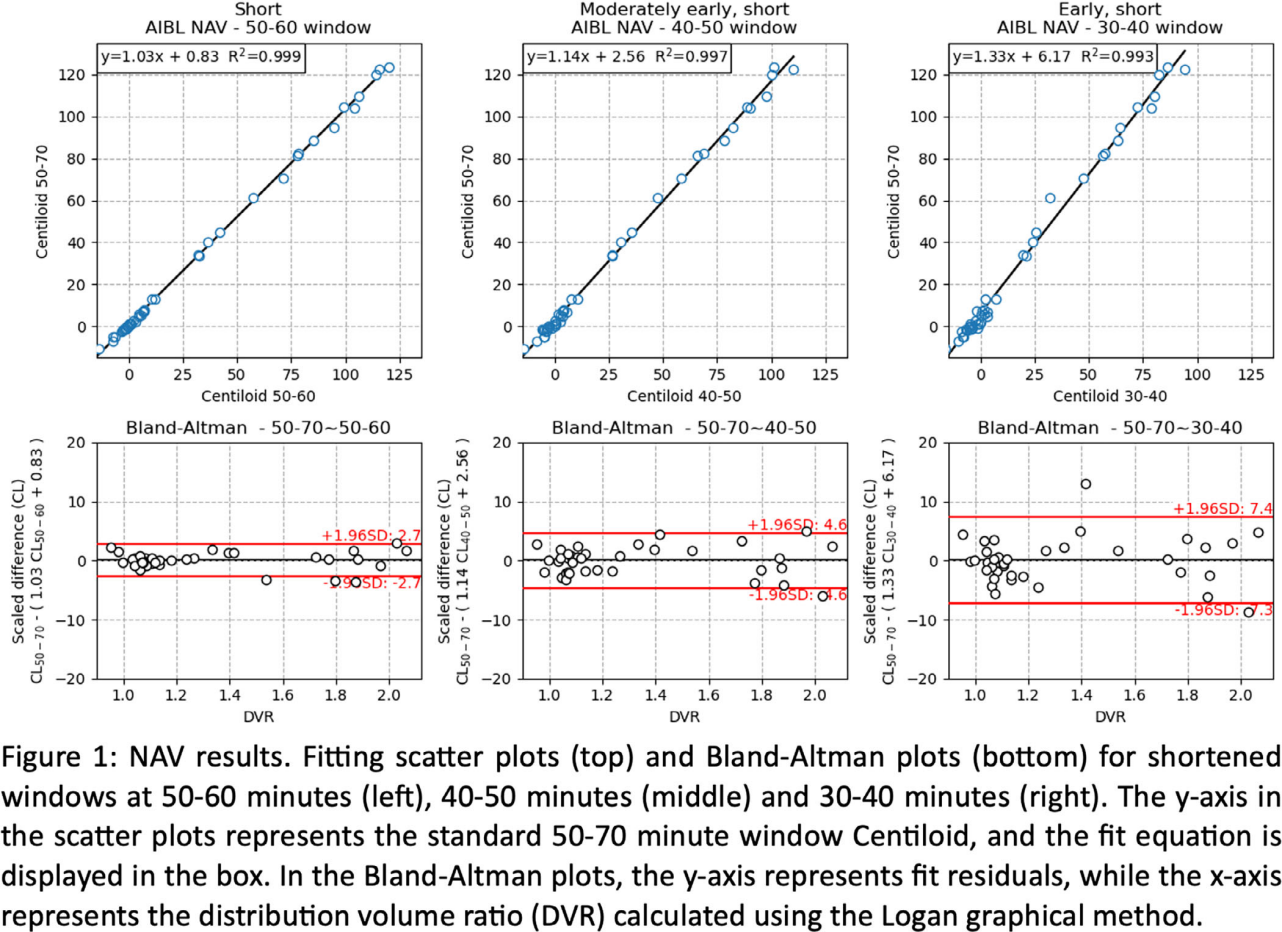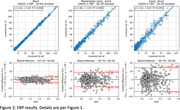# Plausibility of early and short acquisition windows for ^18^F‐NAV4694 and ^18^F‐florbetapir PET to expand clinical throughput

**DOI:** 10.1002/alz70856_104290

**Published:** 2025-12-24

**Authors:** Ashley G Gillman, Pierrick Bourgeat, Jurgen Fripp, Victor L. Villemagne, Graeme O'Keefe, Kun Huang, Tammie L.S. Benzinger, Pamela J. LaMontagne, John C. Morris, Vincent Dore, Christopher C. Rowe

**Affiliations:** ^1^ The Australian e‐Health Research Centre, Commonwealth Scientific and Industrial Research Organisation, Brisbane, QLD, Australia; ^2^ The Australian e‐Health Research Centre, CSIRO, Brisbane, QLD, Australia; ^3^ The University of Pittsburgh, Pittsburgh, PA, USA; ^4^ Austin Health, Melbourne, VIC, Australia; ^5^ Department of Molecular Imaging & Therapy, Austin Health, Melbourne, VIC, Australia; ^6^ Washington University in St. Louis, St. Louis, MO, USA; ^7^ Washington University in St. Louis School of Medicine, St. Louis, MO, USA; ^8^ Washington University in St. Louis, School of Medicine, St. Louis, MO, USA; ^9^ Florey Department of Neuroscience and Mental Health, University of Melbourne, Parkville, VIC, Australia

## Abstract

**Background:**

Monoclonal antibody treatments may lead to increased demand for diagnostic Aβ PET. Simultaneously, high costs and limited reimbursement are pushing clinicians to alternatives such as plasma biomarkers, despite their inability to quantify amyloid burden. Seeking to reduce cost and improve productivity, we assessed the impact of early and short ^18^F‐NAV4694 (NAV) and ^18^F‐Florbetapir (FBP) PET scan protocols on Centiloid quantification.

**Method:**

38 dynamic NAV scans (200±10% MBq) on a Philips Allegro from the AIBL study and 454 dynamic FBP scans (370±10% MBq) on various scanners from the OASIS‐3 study were collected. Centiloid was quantified using the CapAIBL PET‐only software, using the standard 50‐70 minute window and three abbreviated windows of 50‐60, 40‐50 and 30‐40 minutes. A linear association was assumed and estimated between shortened and standard windows, validated with Bland‐Altman analysis. After applying the estimated linear correction, Centiloid error was assessed by mean absolute error (MAE) and 95% range of error (1.96σ).

**Result:**

Figures 1 and 2 (top) depict NAV and FBP, respectively, with fit parameters and metrics summarised in Table 1. Shortened windows correlated strongly with the standard windows (R^2^>0.95), with correlation decreasing for earlier windows. Bland‐Altman analyses revealed residual error was uncorrelated with Amyloid burden and consistent with a linear association for both NAV and FBP (Figures 1 and 2, bottom). NAV at 30‐40 minutes introduced a mean error of 2.7 CL (95% <7.4 CL), or a relative error in SUVR units of 2.1%, comparable to published test‐retest results available for florbetapir (2.4%). This NAV window allows a 50% scan‐time and 40% uptake‐time reduction. For FBP, the 30‐40 minute window yielded higher error (mean 5.4 CL, 95% <14.1 CL), so a 40‐50 minute window (mean 3.3 CL error, 95% <8.5 CL) may be more appropriate.

**Conclusion:**

Acquiring NAV at 30‐40 minutes or FBP at 40‐50 minutes, with linear correction, is unlikely to change clinical management. Thus, 50% scan time reduction and 40% (NAV) or 20% (FBP) uptake time reduction is achievable. Importantly, the tracers have not reached steady‐state at early windows, so accurate timing of the acquisition window is essential.